# The Recent Advances on Liver Cancer Stem Cells: Biomarkers, Separation, and Therapy

**DOI:** 10.1155/2017/5108653

**Published:** 2017-07-27

**Authors:** Yanhong Xiao, Mei Lin, Xingmao Jiang, Jun Ye, Ting Guo, Yujuan Shi, Xuefeng Bian

**Affiliations:** ^1^Taizhou People's Hospital Affiliated to Nantong University, Taizhou, Jiangsu 225300, China; ^2^School of Chemical Engineering and Pharmacy, Wuhan Institute of Technology, Wuhan, Hubei 430000, China

## Abstract

As the third major reason of mortality related to cancer in the world, liver cancer is also the fifth most frequent cancer. Unluckily, a majority of patients succumb and relapse though many progresses have been made in detection and therapy of liver cancer. It has been put forward that in liver cancer, cancer stem cells (CSCs) hold main responsibility for the formation, invasion, metastasis, and recurrence of tumor. Strategies that are intended to target liver CSCs are playing a more and more significant role in supervising the development of liver cancer treatment and assessing new therapeutic methods. Herein, a brief review about molecule markers, signal pathways, separation, and treatment on liver cancer stem cells (LCSCs) is provided in this paper.

## 1. Introduction

As a malignant tumor, the hepatocellular carcinoma (HCC) frequently occurs in the world, which severely threatens the health and life of human beings [1]. It is reported that over six hundred thousand people die of HCC every year. Nonalcoholic obesity liver disease, alcohol abuse, and hepatitis virus are main risk factors leading to liver cancer [2]. Currently, transcatheter arterial chemoembolization (TACE), liver transplantation, surgical treatment, radiofrequency ablation (RFA), radiotherapy, and chemotherapy are traditional therapies of HCC, but these therapies have poor effect for patients at advanced phase, metastasis, or recurrence. It has been popular to seek new approach to treat intermediate, advanced HCC in the past ten years. Recently, the theory of cancer stem cell (CSCs) offers a new idea for prognosis, diagnosis, and treatment for HCC.

According to the theory of CSCs, proposed by Reya et al. [3], only a small amount of cells in tumor tissue, named cancer stem cells, have the ability for indefinite self-renewal and have the multidirectional differentiation potential to generate the heterogeneity of tumor cells. A majority of CSCs are in the G0 stage of the cell cycle and have certain resistance to chemotherapy and radiotherapy, playing an important role in the process of tumorigenesis, metastasis, progression, and recurrence. CSCs have been identified in haematological malignancies and solid tumors such as breast, colorectal, liver, lung, pancreatic, and multiple myeloma. Having been frequently studied in HCC, liver cancer stem cells (LCSCs) have been regarded as the cells with specific stem cell-like features in the liver cancer tissue. Research on LCSC has been rapidly developed in the recent years and encountered with more challenges. Particularly, the identification of targeted therapeutic destruction and LCSC-specific markers is debated most frequently. It is necessary to defy LCSC markers for each tissue. The clarification of signaling functions of LCSC is of great importance to realize accurate identification and diagnosis on the basis of LCSC biomarkers for targeting LCSC, which is bound to enhance the prevention and treatment of LCSC. To better understand and treat LCSC, the markers of stemness and cell fractions associated with metastasis, prognosis, and resistance should be clarified. These markers are of great importance to isolate LCSC and explore the biological feature so as to efficiently target them for therapeutic purposes. Therefore, the current knowledge on putative markers defining LCSC, potential signaling pathways, and therapeutic targets of these markers is summarized. Moreover, the research explores new therapeutic approaches to realize more specific target and eradication of liver LCSC.

## 2. LCSC Markers

A great number of LCSC surface markers such as ALDH, CD133, CD13, CD90, CD44, CD24, OV6, and EpCAM have been found, and new surface markers of LCSC are constantly identified and discussed.

### 2.1. CD133

As a five-transmembrane single-chain glycoprotein to the prominin family with two small and two large intracellular loops, human CD133 is also referred to as AC133 and prominin 1. The role of CD133 as a CSC maker has been documented in varieties of tumor tissues, including gastric carcinoma, lung cancer, liver cancer [4], and colon cancer as well as pancreatic cancer [5]. According to researches, CD133+ liver cancer cells show strong capability of proliferation, differentiation, and self-renewal. According to Suetsugu et al. [6], cells of CD133+ were separated from cell lines of human hepatocellular carcinoma with stem cell-like property or cancer progenitor property. It was reported that CD133+ Huh-7 cells have a greater proliferative ability in vitro than CD133− Huh-7 cells. The study from Rountree et al. [7] showed that CD133+ cells had more resistance to chemotherapy, in comparison with CD133− cells. Tang et al. [8] found that self-renewal, tumorigenesis, and angiogenesis were promoted by CD133+ liver tumor-initiating cells (TICs) through NTS-induced activation of IL-8 signaling cascade. Liu et al. [9] reported that CD133 is significant to monitor the migratory capability of LCSCs, tumor-initiating properties, and the epithelial-mesenchymal transition (EMT) process. It was found by Li et al. [10] that IFN-gamma-induced autophagy was resisted by CD133+ HCC, which may be a mechanism through which immune eradication was resisted by CSCs. Ding et al. [11] found that CD133+ liver cancer stem cells showed resistance to TGF-beta-induced apoptosis. An activated mitogen-activated protein kinase or extracellular signal-regulated kinase pathway is a type of mechanism about resistance to TGF-beta-induced apoptosis in CD133+ cancer stem cells. According to Ma et al. [12], CD133+ HCC cells make contributions to chemoresistance via preferential activation of Bcl-2 and Akt/PKB.

### 2.2. ALDH

Majorly, in the kidney and liver cells of human beings, the metabolism of acetaldehyde and ethanol can be promoted by aldehyde dehydrogenase (ALDH) as a detoxifying enzyme. Involved in the regulation of cell proliferation, ALDH is considered a new cancer stem cell marker in lots of kinds of cancer, including colon, breast, prostate, bladder, and ovary, which is highly expressed in cancer stem cells. It was found by Ma et al. [13] that CD133+ALDH+ cells are more tumorigenic than their counterparts CD133−ALDH− or CD133−ALDH+ in vivo and vitro. In combination of those from their prior work, the data prove that a hierarchical organization exists in HCC with tumorigenic potential in the order of CD133+ALDH+ > CD133+ALDH− > CD133−ALDH−. The population of tumorigenic liver CSC can be featured by ALDH expressed with CD133.

### 2.3. CD44

As a cell surface HA-binding glycoprotein, CD44 is important to tissue remodeling, adhesion of cell-matrix, and cell migration. It has been utilized as a significant marker of solid tumor stem cells. Yang et al. [14] reported the CD90+CD44+ cells showed a more aggressive phenotype than the CD90+CD44− counterpart and developed metastatic lesions in immunodeficient mice's lung. According to Zhu et al. [15], the CD133+CD44+ cells may stand for the true progenitor/cancer stem cells in HCC, which may be a specific target for more efficient treatments and enhance comprehension of progression and initiation in HCC. Williams et al. [16] emphasized on the behavior of CD44-regulating stem cell, including differentiation and self-renewal besides signal transduction and cell-matrix interactions during tumor progression and cell migration. The stem cells of C3A-derived liver cancer were established by OSKM approach (OCT4, SOX2, KLF4, and c-MYC) and termed C3A-induced cancer stem cells (C3A-iCSCs). According to the data, the highly malignant and poorly differentiated tumor cells through maintenance of stemness state were ascribed to nuclear CD44 in liver cancer stem cells.

### 2.4. CD90

As a surface marker of oval cell, a 25–30 kDa glycosylphosphatidylinositol-anchored glycoprotein, CD90, is also referred to as Thy1. According to Yang et al. [14], CD90+ cells from HCC cell lines instead of the CD90^−^ cells showed tumorigenic ability. It was shown by Chen et al. [17] that CD90+ cells have a stronger invasive ability. It was shown that the potential interaction and discrete nature of CD90+ CSCs had specific chemosensitivity and gene expression patterns to molecular-targeted therapy. It was reported that Yang et al. [18] identified CD45−CD90+ CSCs in circulation and tumor tissues. CD45−CD90+ could be utilized to build liver cancer in human beings. It was shown by Jia et al. [19] that in comparison to parental cells (*p* < 0.05), ABCG2 and Oct4 genes were overexpressed in liver CSCs, which are closely related to chemotherapy resistance and highly expressed in enriched CD90+CD133+ liver CSCs. It was presumed that the high recurrence of liver cancer may be ascribed to liver CSCs.

### 2.5. CD13

As a membranous glycoprotein, CD13 is also referred to aminopeptidase N, which is significant to cancer progression, including the invasion, angiogenesis, and proliferation of cell. It was found by Haraguchi et al. [20] that CD13+ cells formed cellular clusters typically in cancer foci and predominated in the G0 stage of the cell cycle. ROS-induced DNA damage was decreased by CD13 after protected cells and radiation/genotoxic chemo stress from apoptosis. On the other hand, the tumor-initiating and self-renewing abilities of dormant CSCs were suppressed by CD13 inhibition. Kim et al. [21] reported that after chemotherapy, CD13+ liver CSC survival in hypoxic lesions may be through the enhanced expression of aminopeptidase N or CD13. In addition, it was shown that there was an EMT-related decrease in elevation of ROS and the expression of CD13 was important to the survival of CSCs. It was shown by Yamashita et al. [22] that the proportion of cells in G0/G1 was reduced by ubenimex, an inhibitor of CD13. Through the increase of intracellular ROS levels and apoptosis, the therapeutic effects of 5-FU, CDDP, and DXR can be improved by ubenimex.

### 2.6. OV6

OV6 can be considered as a marker of hepatic oval cells. Roskams et al. [23] reported that reactive ductules and intermediate hepatocyte-like cells originate at least partly from differentiation and activation of “progenitor cells.” It was put forward that OV6 in human liver can recognize cells with a progenitor stem cell-like phenotype, which has the ability to differentiate into OV6-positive ductular cells or lobular hepatocytes. Yang et al. [24] found that in comparison to OV6- tumor cells, these OV6+ HCC cells had more capabilities to form tumor in vivo, showing a more considerable resistance to standard chemotherapy. According to Yang et al. [25], cells of OV6+ HCC might stand for a subpopulation of TICs with metastasis potential and augmented invasion, which makes contribution to metastasis and progression of HCC. Furthermore, the CXCR4/SDF-1 axis offers therapeutic targets to eliminate liver TICs. In current days, as one of the LCSCs surface markers, further studies on OV6 are needed.

### 2.7. EpCAM

As a single transmembrane glycoprotein in a family of adhesion molecules, epithelial cell adhesion molecule (EpCAM) is also referred to as CD326. According to data [26], EpCAM functioning as a Wnt-beta-catenin signaling target gene may be utilized to promote prognosis of HCC by facilitating efficient stratification of patients with predicted pharmacologic responses to Wnt-beta-catenin signaling antagonists. According to the results [27], the development and invasiveness of HCC is described by a subset of EpCAM+ cells to open a new avenue for eradication of HCC cancer cell by targeting beta-catenin or Wnt signaling components, like EpCAM. According to Terris et al. [28], it was shown by pathway analyses and gene expression that the subtype of EpCAM+ AFP+ HCC was featured by progenitor/hepatic stem cells. Hepatic cancer stem cell-like traits such as the capabilities to differentiate and self-renew were shown in the cells of fluorescence-activated cell sorting isolated EpCAM+ HCC. According to Yamashita et al. [29], the study of gene expression on sorted cells showed that EpCAM+ cells had features of epithelial cells. According to clinicopathological study, the presence of EpCAM+ cells was related to high serum alpha-fetoprotein (AFP) and poorly differentiated morphology.

### 2.8. Side Population Cells

As a member of the transporters of ATP-binding cassette (ABC), ABCG2 was frequently shown in stem cells. Furthermore, conferring the side population phenotype, ABCG2 is considered a common marker of stem cells and is crucial to the promotion of the stem cell phenotype maintenance and stem cell proliferation. It was shown by Zhou et al. [30] that as a crucial determinant of the SP phenotype, ABCG2/Bcrp1 gene expression might make stem cells from varieties of sources. The side population (SP) cells were adopted to establish cell lines of hepatocellular carcinoma (HCC) for detection of subpopulations as cancer stem cells and for identification of the role in tumorigenesis. SP cells were detected in PLC/PRF/5 and Huh7 cells instead of in Huh6 and HepG2 cells amongst four cell lines which had been studied. In comparison with those of non-SP cells, antiapoptotic properties and high proliferative potential were shown in SP cells. Jia et al. [19] found that ABCG2 and Oct4 are expressed in enriched CD90+CD133+ liver CSCs which had close relation to chemotherapy drug resistance. It was presumed that high recurrence in liver cancer may be ascribed to liver CSCs. Zhang et al. [31] discovered that ABCG2-positive cells have a strong tumorigenicity. The malignant behaviors were reduced by downregulation of ABCG2 whereas the ability of proliferation, doxorubicin resistance, migration, and invasion potential was improved by upregulation of ABCG2. The expression of ABCG2 has been considered a factor of poor prognosis.

### 2.9. CD24

As a heavily glycosylated and small mucin-like cell surface glycoprotein, CD24 is expressed in progenitor or stem cells. CD24+ HCC cells have a great impact on clinical outcome of patients, playing an important role in the self-renewal, differentiation, maintenance, and metastasis of tumors [32]. As a functional liver T-IC marker, CD24 uses STAT3-mediated NANOG regulation to drive T-IC genesis. It was demonstrated by Liu et al. [33] that as a highly conserved helix-loop-helix transcription factor, Twist2 is important to self-renewal of augmented liver cancer stem-like cell in a CD24-based manner. The pathway of Twist2-CD24-STAT3-NANOG might be crucial to the regulation of self-renewal of liver cancer stem-like cell.

### 2.10. DLK1

Though expressed in fetal liver, DLK1, Delta-like 1 homolog, was not found in adult and neonatal liver in rats and mice. Huang et al. [34] reported that proliferation of SMMC-7721 cells, a HCC cell line, could be greatly promoted by exogenous DLK1 while colony formation, cell growth, and tumorigenicity of Huh-7, Hep3B, and HepG2 cells can be greatly inhibited by the suppression of endogenetic DLK1 via RNA interference. According to these data, as an imprinted gene, DLK1 can be greatly upregulated in HCC and make contribution to the tumor oncogenesis because of certain epigenetic events. It was found by Li et al. [35] that, expressed in malignancies, Delta-like 1 homologue (DLK1) enhancing the tumourigenicity and stemness of cancer cell potentially becomes a molecule target for treatments against cancer stem or progenitor cells.

### 2.11. K19

As widely known, keratin 19 (K19) is a marker of strong invasion and poor prognosis in the hepatocellular carcinoma (HCC) of human beings. Compared to K19-negative HCC, K19 was most frequently related to a poor prognosis as well as mRNA- and EMT-related proteins and more frequent main vessel invasion was shown in K19-positive HCC [36]. It was found by Bae et al. [37] that as an independent prognostic factor which could be hardly found in dysplastic nodules (DNs), the expression of K19 commonly occurred in small progressed HCC. According to their findings, the expression of K19 might be a characteristic of carcinoma cells during the progression in certain HCC. It was found by Kawai et al. [38] that K19+ cells had high proliferation capacity and 5-fluorouracil resistance in vitro. According to the findings, K19+ cells were involved in the activation of Smad/TGFb signal and EMT. These properties could be suppressed by TGFbR1 inhibitor or K19 knockdown. As a new CSC marker related to Smad/TGFb signal and EMT, K19 was considered as a sound therapeutic target for the inhibition of TGFbR1.

### 2.12. C-kit

As a receptor protein of transmembrane type III with intrinsic tyrosine kinase activity to make human embryonic stem cells, human C-kit is also named as stem cell factor receptor (CD117). Besides, having been used to recognize human hematopoietic and progenitor cells/hepatic stem, human C-kit can maintain the undifferentiated status of stem cells. Fujio et al. [39] pointed out that C-kit may be a significant member of the receptor systems or growth factor related to the biology of liver stem cells and that the development of bile ducts includes the stem cell c-kit signal/factor transduction system. According to the findings [40], cells with CD34 or markers c-kit in human liver have the ability to differentiate from lineage of biliary epithelial cell and thus may show biliary epithelial progenitor cells of human beings. It was found by Lee et al. [41] that the C-kit+ sinusoidal cells are important to progression and angiogenesis of hepatitis B virus-related HCC in human beings.

### 2.13. SALL4

As homeotic genes for embryonic development, the spalt (sal) family is a class of evolutionarily conserved genes which have been identified in Drosophila originally. The expression of sal-like 4 (SALL4) in vertebrates is enriched in adult stem-like/stem and embryonic cells [42] specifically. As a master regulator, SALL4 makes contribution to cell stemness in the growth of tumor and biological development. As a subtype of hepatocellular carcinoma (HCC) markers, SALL4 is related to the prognosis of liver cancer [43]. It was shown by Han et al. [44] that as a new biomarker in the prognosis of HCC patients, high levels of SALL4 serum tended to poor prognosis, recurrence, and low survival rate. According to Yakaboski et al. [45], as an oncofetal protein, silenced in the adult liver and expressed in the human fetal liver, SALL4 is reexpressed in a subgroup of patients with an unfavorable prognosis and hepatocellular carcinoma. According to the result of gene expression analysis, the overexpression of metastatic and proliferative genes in SALL4-positive hepatocellular carcinomas enriches the progenitor-like gene.

### 2.14. Others

As a 90-kD cell surface glycoprotein of the immunoglobulin superfamily, intercellular adhesion molecule 1 (ICAM-1) is generally believed to realize HCC metastasis. Liu et al. [46] pointed out that ICAM-1 is conductive to effectively treat cancer as a potential CSC marker. Moreover, ICAM-1+ HCC has been proved to be highly sphere forming with a great tumorigenic capability and can increase the expression of stemness-related genes compared with their ICAM-1− counterparts. MAb 1B50-1 binding to *α*2*δ*1 + isoform 5 selectively targets LCSCs in human HCC cells. The existing research indicates that tumors can be initiated by 1B50-1+ cells. Binding to a subpopulation of HCC cells, 1B50-1 hereafter is termed as *α*2*δ*1 + cells which presents the stem cell-like properties, such as the expression of stem cell, associated genes (OCT4, SOX2, NANOG, and BMI1), higher self-renewal ability, increased invasiveness, and the ability to generate both *α*2*δ*1+ and *α*2*δ*1- cells [47]. Recently, Nanog gene which is a member of the homeobox family of DNA binding to transcription factors has been identified in a screen for genes promoting pluripotency. Nanog directly gives rise to the deterioration of solid tumors. According to the accumulated evidence, it has been found that the levels and expression of Nanog were upregulated in ovarian, breast, gastric, colorectal, head and neck squamous cell, hepatocyte, lung, and prostate carcinomas. This suggests that Nanog plays a potential role in tumorigenesis. Nanog + Huh7 cells have CSC properties compared with Nanog − Huh7 cells, such as higher chemotherapy resistance, tumor sphere formation, and self-renewal ability [48].

## 3. Signaling Pathways

A great deal of research has proved that Notch, Wnt/*β*-catenin, hedgehog, and TGF-*β* signaling network are implicated in maintaining the tissue homeostasis by adjusting the self-renewal ability of normal stem cells as well as proliferation or differentiation degree of progenitor cells. Particularly, the importance of Wnt/*β*-catenin and hedgehog signaling pathways for embryogenic development has been verified in the acquisition of EMT and the biology of CSCs. The transformation to CSC is caused by the breakage of the signaling network for normal stem cells. Because of genetic alteration or epigenetic change of stem cell signaling-related genes, CSC is caused by self-renewal potential in progenitor cells. The dysregulation of Notch, Wnt/*β*-catenin, hedgehog, and TGF-*β* signaling pathways in CSCs from numerous human tissues or organs should be investigated systematically so as to better understand CSCs and the corresponding role in carcinogenesis. During hepatocarcinogenesis process, dysregulation of signaling pathways was found and the signaling pathways of Wnt, TGF-*β*, hedgehog and Notch have been studied extensively.

### 3.1. Wnt Signaling Pathway

As classical and important regulator of stem cells, Wnt has increased the possibility that mediated by Wnt signalling in progenitor cells and stem cells. The subverting of self-renewal which is strictly monitored may lead to cell malignant proliferation. According to Miyoshi et al. [49], because of amino acid substitutions at their interstitial deletions or neighboring codons involving exon 3 or at potential threonine/serine phosphorylation residues, the accumulation of beta-catenin could make contribution to hepatocellular carcinogenesis. According to the results [50], the signaling pathway of Wnt by beta-catenin mutation makes great contribution to the hepatocellular carcinogenesis related to the infection of HCV. It was discovered by Wong et al. [51] that hepatocarcinogenesis was related to deregulation and beta-catenin mutation. Beta-catenin overexpression of nonnuclear type tended to have prognostic and pathologic importance. The progression and genesis of HCC can be affected by beta-catenin through the signal transmission pathway of Wnt. Simultaneous determination of beta-catenin, HBsAg, and AFP is significant to metastatic, diagnosis, and clinical staging of HCC [52]. The regulation of angiogenesis, metastasis, and infiltration of HCC may be ascribed to the beta-catenin/Wnt signaling pathway through the regulation of the angiogenic factor regulation. The beta-catenin/Wnt pathway is an attractive target to develop new rational treatment for HCC, an illness for which few therapies are available in current days.

### 3.2. Notch Signaling Pathway

An ancient mechanism of cell interaction was defined by Notch signaling, playing a crucial role in the development of metazoan. Cell apoptosis, proliferation, and differentiation are all related to Notch activity which may offer a development tool for the formation and morphogenesis of organ. According to Gao et al. [53], Notch signaling may be involved in the hepatocellular carcinoma development and the expression of Notch receptors was deregulated. Activated in HCC samples of human beings, Notch signaling as a biomarker of response to inhibition of Notch in vitro [54] facilitates hepatocarcinogenesis in mice.

### 3.3. The Hedgehog (Hh) Signaling Pathway

The pathway of hedgehog (Hh) was proved to be a contributor to the progression and carcinogenesis of different types of tumor. Hedgehog signaling might be activated in some tumors of HCC [55]. According to data, the pathway of hedgehog (Hh) is significant to the formation and invasion of HCC. The new HCC treatment may consider blockade of the Hh signaling pathway as a potential target [56]. Hepatocarcinogenesis and liver fibrosis can be promoted by the activation of Hh pathway.

### 3.4. TGF-*β* Signaling Pathway

Transforming growth factor-beta (TGF-beta) and TGF-beta-related proteins have been considered as crucial regulators of renewal and differentiation of stem cell. The TGF-beta 1 expression was related to the histological differentiation of hepatocellular carcinoma cells. To summarize, in human HCC, the malignant potential can be accelerated by distortion of autocrine TGF-beta signals through VEGF and PAI-1 production and enhancement of cell growth. TGF-beta gene in HCC [57] may be against the progression of tumor by monitoring the occurrence of spontaneous apoptosis and the surviving protein expression in tumors. It was reported previously that inhibition of TGF-beta receptor-dependent growth was shown in HCC cells in response to TGF-beta. Furthermore, there is decreased TGF-beta receptor II in HCC correlating with shorter time-to-recurrence and intrahepatic metastasis. It suggests the role that TGF-beta signaling plays in suppression of tumor. Oppositely, relating to malignant potential, the overexpression of TGF-beta in HCC suggests a role in the promotion of tumor. Enhanced formation of stroma is a characteristic of advanced HCC, and TGF-beta promotes the proliferation of stromal fibroblasts as well. Though it inhibits hepatocyte proliferation, transforming growth factor-beta (TGF-beta) induces the fibrosis in hepatic cirrhosis; in relation to aggressive characteristics of HCC, like IM, the expression of TGFBR2 (transforming growth factor-beta receptor II) may stand for an immunohistochemical biomarker for detection of aggressive HCC.

## 4. LCSC Separation

To recognize and separate CSC so as to research their qualities accurately is quite significant. In order to characterize CSCs, various analytical approaches and skills have been adopted. Frequently used approaches for identifying and separating CSCs contain molecular, functional, image-based, cytologic arrangement, and percolation methods, the usage of various membrane markers and xenografts. LCSCs can be recognized and separated through 4 major methods: separation by flow cytometry on the basis of CSC-specific cell membrane markers [58, 59]; recognition of side population (SP) phenotype excluded by Hoechst 33342 assay [60]; decision of capability to develop as flowing balls in serum-free medium [61, 62]; and evaluation of aldehyde dehydrogenase (ALDH) liveness [63].

Side population (SP) cellular arrangement was originally used to recognize haematopoietic stem cells and has been adopted in different tissues and organs to fertilize stem cell fraction. SP cells are recognized by their own capability to pass through Hoechst 33342 dye by adenosine triphosphate- (ATP-) binding cassette (ABC) membrane transporter. More currently, SP cells have also been adopted to try to separate stem cell-like components from cancer cells. This method appears to be logical and estimable, as it is recorded that many cancers, containing HCC, highly expressed ABC transporters and intimately have a strong role in multidrug resistance. Cell-side population (SP) arrangement approach adopting Hoechst 33342 dye can have effects of cytotoxic on non-SP cells. Other dyes, like SYTO-13 or rhodamine 123 (Rh123), are also likely to be adopted [64]. An association of Rh123 and Hoechst 33342 has been adopted to fertilize hemopoietic stem cells. CSCs comprise different cell-specific markers which are distinct from non-CSC populations. These markers are part of the class of membrane proteins. Another approach is to use MACS or FACS on the basis of the separation and abundance of positive selection of CSCs by membrane markers for example EpCAM, CD44, CD90, and CD133. Fluorescence-activated cell sorting (FACS) is another method of separation that is carried out by fluorescent dyes in indirect or direct immunofluorescent staining. Fluorescent pigments can be immediately combined with either primary or secondary antibodies. In general, FACS isolation employs fluorescent dyes along with various emission wavelengths. Magnetic-activated cell sorting (MACS) is found on superparamagnetic and biodegradable microspheres connected to a particular monoclonal antibody (mAb), which permits improvement of cells expressing the wanted antigen. Even though MACS is easier and demands less sophisticated device than FACS, it is one parameter and cannot separate cells by numerous markers at the same time.

## 5. LCSC Treatment

Even though ionizing radiation and chemotherapy wipe out tumor cells in the proliferative cell cycle, CSCs have native resistance to these therapies. Intervention with self-renewing, subsistence, and niche characteristics of CSC is a feasible tactic for targeted treatment.

To inhibit CSC-specific approaches is a hopeful medicinal treatment. It was suggested by Kreso et al. [65] that the self-renewing of the CSC function of the colorectal is extremely relied on the PcG protein BMI1. The pharmacological damage of EZH2 (the main constituent of PRC2) by the S-adenosine homocysteine hydrolase inhibitor, 3-deazapatocin A (DZNep), undercuts the self-renewing and tumor initiation abilities of some cancers, HCC included [66]. Clinical tests are required to check whether these drugs are clinically useful to eliminate liver CSC. Disorder of epigenetic mechanisms, which contains histone modifications and DNA methylation, is intimately related to cancer growth and advancement. Cumulative proofs indicate that the effectiveness of epigenetic drugs in eliminating CSC in HCC, Zebularine, a DNA methyltransferase (DNMT) inhibitor, was displayed to reduce CSC features in high-density-grown HCC cells, like self-renewing and tumorigenicity [67]. In summary, epigenetic treatment with inhibitors of DNMT and/or HDAC [68, 69] is likely to be a hopeful method for eliminating CSC in HCC. Monoclonal antibodies aiming at CSC-specific antigens seem to be a hopeful method for eliminating CSC [70]. The effectiveness against CD133, CD13, and EpCAM is reported for eliminating hepatic CSC [71]. Overexpression of HNF4A, a major adjuster of hepatocyte distinction, led to a reduction in the amount of tumorigenic CD90+ and CD133+ cells [72], while at the same time bringing about the cells to lose the tumorigenicity through distinction of the induced grouped population. Traditional cytotoxic agents cast a significant part in decreasing the volume of tumor, in view of the fact that promptly proliferating cells, rather than CSC, often make contributions to a large portion of the tumor. The combination of traditional cytotoxic agents and the CSC-targeted drugs described above seems to be one of the most hopeful tactics for cancer therapy.

Cancer stem cells (CSCs), tumor-initiating cells (TICs), are quite hard to eliminate with traditional cancer therapies, for example, chemotherapy and radiotherapy. Therefore, the survival of the remaining CSCs is considered to be the start of tumor recurrence, drug resistance, and distant metastasis, which is an important clinical issue in the actual therapy of cancer. As a result, there is an urgent need for new methods of cancer treatment to deal with this clinical demand. Graphene and derivatives thereof are renowned, comparatively inactive and possibly poisonless nanomaterials which come into being steady dispersoids in all kinds of solvents. Right here, we display that graphene oxide (of both large and small schistose magnitudes) can be employed selectively to restrain tumor stem cell proliferation across numerous tumor kinds. The proofs provided by us show that GO performs an astonishing influence on CSCs by restraining some critical signal transduction pathways (Notch, WNT, and STAT signaling) and thus eliciting CSC variation. Therefore, graphene oxide is likely to be an efficient poisonless treatment tactic for eradicating cancer stem cells through variation-founded nanotherapies [73]. The therapy of hepatocellular carcinoma (HCC) or hepatic injuries has been hampered by a shortage of effective drug delivery. Even under the assistance of nanoparticles or other composition delivery drugs, most of the dosages are still separated in the reticuloendothelial system. Superparamagnetic iron oxide coating gold nanoparticles (SPIO@AuNPs) is a poisonless, magnetic resonance- (MR-) positive contrast agent which could produce heat when radiated with near-ir lasers. The SPIO@AuNP-loaded AD- MSCs demonstrated a hopeful therapy for impaired liver and HCC. The iRGD (internalized Arg-Gly-Asp peptide) - gemeled DSPE-PEG2000 nanomicelles (M-SAL-iRGD) displayed importantly enhanced cytotoxic influence to both nontargeted M-SAL (salinomycin-containing DSPE-PEG2000 nanomicelles) and salinomycin in CSCs and liver cancer cells. The antitumor trials and tissue distribution in mice with liver cancer xenografts demonstrated the ranking tumor-penetrating effectiveness and antitumor liveness of M-SAL-iRGD. M-SAL-iRGD stands for a possible potent antihepatoma drug [74]. Disulfiram (DS) is an antialcoholic drug that exhibits quite intense cytotoxicity in a lot of cancer kinds. The DS-PLGA (poly lactic-co-glycolic acid- (PLGA-) encapsulated DS) owns extremely well-pleasing capsulation potency, content of drug loading, and in vitro controlled release ratio. It proved as well extremely hopeful anticancer effectiveness and antimetastatic role in mouse liver cancer models. The research is likely to result in rearrangement of DS into liver cancer therapy [75].

## 6. Clinical Implications of LCSCs

In recent days, various markers were applied in the identification of LCSCs. Later, a number of deregulated molecular pathways had been found in LCSCs. In-depth research showed that the markers may make contribution to diagnosis, prognosis, and treatment in HCC patients. It was found by Yang et al. [18] that CD45−CD90+ cells could be found in all the samples of tumor, yet none in the parallel (normal and cirrhotic nontumorous livers). Besides, CD45−CD90+ cells could be detected in 90% of blood samples from patients with liver cancer, yet not in cirrhosis patients or normal subjects. CD45−CD90+ can serve as a target to diagnose and treat malignancy and as a marker for liver cancer in human beings according to the identification of CD45−CD90+ CSCs in circulation and tumor tissues. According to Piao et al. [76], the liver cancer cells of CD133 expression have radioresistance and antiapoptotic properties which can enhance anticancer therapies, such as radiotherapy/chemotherapy of HCC. According to Song et al. [4], reactivated CD133-positive cells can be commonly found in HCC. In addition, the higher stage of HCC tumors, the more expression of CD133, the poorer prognosis for patients.

The progress that targeted treatments against LCSC is promoted by study on the signaling pathways in HCC pathogenesis. It was found that the invasiveness and proliferation of hepatocellular carcinoma, including the pathway of beta-catenin/Wnt could be suppressed by Biejiajian Pills [77], 1118–20 [78], and destruxin B [79]. Furthermore, they may be used to treat HCC in the future. HCC cell growth can be inhibited by thymoquinone (TQ) by inducing apoptosis and cell cycle arrest, which may be achieved by repression of the signaling pathway of the Notch. TQ is a potential therapeutic or preventive agent for HCC [80]. It was reported [81] that sulfatase 1 (SULF1) played an important role in the progression of HCC tumor via augmentation of the TGF-beta pathway. SULF1 may be a new target in searching drug treatment of HCC and may be a potential biomarker for the progression of tumor.

## 7. Conclusions

The theory of CSCs in liver cancer of human beings can be supported by lots of evidences. The isolation and identification of LCSCs can be considered as a basic study. A number of markers, including CD13, CD44, CD24, CD133, CD90, EpCAM, and SP cell can be used to identify LCSCs. However, many critical problems still need to be found and solved. There is still not a consensus of an “international” marker for LCSCs. The amount of CSCs is different based on cell lines, and not all the isolated cells by CSC markers were CSCs. Besides, some of the pivotal markers which are important to CSCs can be shared by normal stem cells as well. Therefore, normal stem cells can be affected by drugs which target these markers in a negative way. Lots of data have shown that the deregulation of many signaling pathways are significant to CSC self-renewal, which can potentially be the targets for treatment. Further researches on the differential signaling pathways between CSCs and the amount of normal stem cell may decrease the negative impacts of drugs on the regeneration of normal tissue. Current study emphasizes on a novel generation of anticancer drugs which target different amounts of CSCs selectively. As researches on surface markers and relevant signaling pathways as well as molecular biology develop, new tumor-specific therapies may be applied to cure liver cancer patients in the future.
